# Citrus Pectin Supplementation Alleviated Hepatic Lipid Accumulation through Gut Microbiota Indole Lactic Acid Promoting Hepatic Bile Acid Synthesis and Excretion

**DOI:** 10.7150/ijbs.116929

**Published:** 2025-07-28

**Authors:** Zhijun Pan, Xinyuan Jin, Qing Li, Yuqing Zhou, Yupeng Zeng, Xin Wang, Yufeng Jin, Yu Chen, Dan Li, Wenhua Ling

**Affiliations:** 1 Department of Nutrition, School of Public Health, Sun Yat-sen University, Guangzhou, China, 510080.; 2 Guangdong Provincial Key Laboratory of Food, Nutrition and Health, Guangzhou, China, 510080.

**Keywords:** citrus pectin, gut microbiota, Indole-3-lactic acid, hepatic lipid accumulation

## Abstract

Metabolic-associated fatty liver disease (MAFLD) represents a critical global health challenge. A few studies have suggested that citrus pectin may confer protective effects against MAFLD; however, the underlying mechanism remains unclear. The gut microbiota and its metabolites strongly contribute to MAFLD regulation by the gut‒liver axis. The present study explored the influence of pectin intervention on liver lipid accumulation in high-fat and high-sugar diet-fed mouse models. Pectin supplementation alleviated hepatic lipid accumulation and substantially restructured the gut microbial communities, particularly enhancing the proliferation of *Akkermansia muciniphila* (*A. muciniphila*) and *Escherichia coli* (*E. coli*), which subsequently increased indole-3-lactic acid (ILA) production. Mechanistic investigations revealed that ILA upregulated hepatic CYP7A1 and FXR-BSEP expression, stimulating hepatic bile acid biosynthesis and biliary excretion to alleviate liver steatosis. Results of previous fecal microbiota transplantation (FMT) and antibiotic-mediated microbial dysbiosis studies have confirmed the microbiota-dependent nature of the therapeutic effects of pectin. Furthermore, the administration of exogenous ILA has been demonstrated to be an effective intervention for the rescue of metabolic dysregulation in dysbacteriosis mouse models. This work delineated an unrecognized dietary pectin-microbiota-ILA-hepatic bile acid synthesis and excretion regulatory axis for the improvement of MAFLD.

## 1. Introduction

MAFLD has reached epidemic proportions globally, with the prevalence increasing alongside rising rates of obesity, type 2 diabetes, and metabolic syndrome 1. It is characterized by excessive lipid accumulation in the liver, driven by an imbalance between lipid uptake, synthesis, and export, leading to hepatic steatosis and metabolic dysfunction 2. Liver steatosis due to the dysregulation of cholesterol metabolism is also a significant risk factor for the pathogenesis of MAFLD, and reducing hepatic cholesterol levels has proven to be an effective therapeutic approach. The cholesterol metabolic processes within the liver are involved mainly in endogenous synthesis and efflux, including bile acid synthesis and excretion. Cholesterol is used as a raw material for the synthesis of bile acids by the bile acid-synthesizing rate-limiting enzyme CYP7A1. Bile acids are subsequently excreted into the bile ducts by the bile salt efflux pump BSEP. In addition, hepatic cholesterol can be directly flowed out to the bile and intestinal lumen through AGCG5/8 regulation and subsequently to feces. Several studies have demonstrated the efficacy of strategies designed to promote cholesterol conversion to bile acid and excretion to prevent hepatic lipid accumulation and alleviate MAFLD 3-5. However, few studies have focused on the promotion of hepatic bile acid synthesis and efflux through the activation of hepatic CYP7A1 and BSEP, with the objective of reducing hepatic cholesterol levels and consequently ameliorating MAFLD.

Importantly, FXR is a critical nuclear receptor that plays a pivotal role in the regulation of hepatic lipid accumulation. Numerous studies have demonstrated that the activation of hepatic FXR significantly improves MAFLD. Furthermore, both CYP7A1 and BSEP are regulated by hepatic FXR. FXR has the capacity to directly activate the proximal promoter of BSEP, thereby increasing its expression. Concurrently, the activation of hepatic FXR can upregulate the expression of the small heterodimeric chaperone SHP, which in turn inhibits the expression of CYP7A1. Notably, the regulation of CYP7A1 by FXR is indirect and can also be influenced by other factors, such as gene polymorphisms, diet, hormones, and cytokines. Consequently, in the presence of dietary intervention, the regulation of CYP7A1 by FXR has different outcomes that may not be consistent with other studies 3,4. Therefore, the potential of FXR activation to promote hepatic bile acid synthesis and efflux through the regulation of CYP7A1 and BSEP as a strategy to ameliorate MAFLD warrants further investigation.

Currently, targeting the gut microbiota and its metabolites through dietary interventions offers a promising therapeutic strategy to modulate microbial composition and function, thereby alleviating MAFLD and its associated metabolic disturbances 6. Several cross-sectional studies have shown that overgrowth of Bacteroidetes may play a key role in MAFLD development 7. Wong et al. 8 reported that decreased intrahepatic triglyceride levels in patients with MAFLD were related to a reduction in Firmicutes and an increased abundance of Bacteroidetes. Recently, *A. muciniphila* has received significant attention as a beneficial bacterium with therapeutic potential for treating metabolic disorders, including obesity, diabetes, and MAFLD. The latest study showed that *A. muciniphila* intervention in mice fed a high-fat diet reshaped the gut microbiota. It also upregulated the mRNA expression levels of hepatic genes involved in bile acid synthesis (CYP7A1, CYP8B1, and CYP27A1) and transport (TGR5, BSEP, and MRP2/3), thereby improving MAFLD 9.

The role of intestinal metabolites as key intermediary factors in targeting the liver directly by the portal vein to improve MAFLD has also received increased attention. A wide variety of intestinal metabolites, including tryptophan metabolites produced by intestinal flora (indole and its derivatives), have been reported to be negatively correlated with MAFLD in a population-based study 10, suggesting that indoles and their derivatives may play potentially protective roles in regulating MAFLD. ILA has received considerable attention in recent studies due to its biological activity. Qian et al. 11 demonstrated that ILA can activate AHR, thereby improving gut health and neuroinflammation. Additionally, ILA has been shown to alleviate colorectal tumorigenesis through epigenetic regulation of CD8+ T cell immunity 12 and to alleviate obesity through epigenetic regulation of bile acid metabolism by CYP8B1 13. Therefore, ILA has therapeutic potential for MAFLD that needs to be investigated.

The intricacies inherent in the pathogenesis of MAFLD have led to a paucity of pharmacological treatment options. Consequently, there has been an increasing focus on prevention and treatment strategies based on dietary interventions. The augmentation of dietary fiber intake is widely regarded as a pivotal daily dietary intervention strategy for individuals with MAFLD. Pectin, a soluble macromolecular dietary fiber abundantly present in fruits and vegetables, has many beneficial effects on human health, including attenuating atherosclerosis, anti-obesity, and anti-inflammatory effects 14. However, the mechanisms underlying these health effects remain unclear. Due to its indigestibility, pectin has been shown to exert prebiotic effects by promoting the growth of beneficial gut bacteria and regulating the production of microbial metabolites 15-17. Preliminary studies have investigated the effects of pectin on microbial communities and their final metabolite short-chain fatty acids while neglecting the effects on other gut metabolites 14,18. Although the latest cohort studies have demonstrated a positive association between fiber intake and serum ILA and IPA levels 19-21, few studies have focused on the impact of pectin supplementation on gut tryptophan metabolites, including but not limited to ILA, in a mouse model of MAFLD.

In this study, we demonstrated that citrus pectin intervention could enhance glucose‒lipid metabolism and reduce hepatic lipid accumulation in mice induced by a Gubra‒Amylin NASH (GAN) diet (20% fat, 20% fructose, and 2% cholesterol). Moreover, pectin intervention has been shown to alter the structure of the intestinal flora and promote tryptophan metabolism in the intestine, increasing the levels of the tryptophan metabolite ILA in the feces and serum of mice. We screened and identified ILA as a critical mediator of pectin-induced MAFLD and metabolic dysfunction improvement. It was found to upregulate hepatic CYP7A1 and FXR-BSEP expression, promoting hepatic bile acid synthesis and excretion to the gallbladder, leading to a reduction in hepatic total cholesterol (TC) levels.

## 2. Materials and Methods

### 2.1. Molecular weight and structural characterization analysis of pectin

Two milligrams of the pectin sample from Sigma-Aldrich (Cat# P9135, USA) was dissolved in 2 mL of deionized water. The mixture was filtered through a 0.45 μm membrane and injected into a gel permeation chromatography (GPC) system. The chromatographic conditions were as follows: Waters 1414 refractive index detector; Waters Ultrahydrogel 1000 column (7.8*300 mm); 30℃ column temperature; and 100 mM sodium nitrate aqueous solution (0.5 mL/min) mobile phase.

For Fourier transform infrared spectroscopy (FTIR), the solid sample was ground in an agate mortar, mixed with KBr powder at a 1:100 ratio, and pressed into a thin film under vacuum. The FTIR range is 0-4000 cm⁻¹. Analyses were conducted with support from the Testing Center of Sun Yat-sen University. The mehydroxydiphenyl approach was used to measure the galacturonic acid content.

### 2.2. Animal study

All animal procedures adhered to the ARRIVE guidelines and were approved by the Animal Ethics Committee of Sun Yat-sen University (No. 2022-039). Six-week-old male C57BL/6J mice were purchased from Guangdong Medical Laboratory Animal Centre and housed in ventilated cages under controlled conditions: 23±3°C, 40-60% humidity, a twelve-hour light/dark cycle, and ad libitum access to food and water (≤5 mice/cage). Fresh food and water were changed every three days, and the mice were observed weekly to record food intake and body weight.

**Experiment 1**: In the pectin intervention study, fifty-six 6-week-old male mice were equally randomized into seven groups and fed a chew diet or GAN diet with varying doses of pectin: (A) control, (B) C5P, (C) GAN, (D) G0.5P, (E) G5P, (F) G10P, or (G) G20P. Groups A-B were fed a chew diet with 10% kcal fat (D12450J, research diet, USA) or a chew diet with 0.5% (w/w) pectin. Groups C-G were fed a GAN diet (20% fat, 20% fructose, and 2% cholesterol; D9100310, Research Diets, USA) or a GAN diet supplemented with 0.5%, 5%, 10%, or 20% (w/w) pectin.

**Experiment 2**: Thirty male mice (6-week-old) were split into three groups for an FMT study: F-GAN, F-G5P, and F-G5PH. All groups were fed the GAN diet for 8 weeks. To eliminate preexisting gut flora, 17 mmol/L PEG3350 was added to the drinking water, and all mice fasted overnight before receiving FMT. Fecal slurry was prepared by harvesting feces from mice fed the GAN or G5P diet for 8 weeks. The feces were resuspended in 0.9% sterile sodium chloride solution at a dilution rate of 200 mg of feces per 1 mL, filtered through a 100 μm strainer, and later transplanted. Each mouse then received 200 µL of the designated FMT through gastric lavage every other day from week 4 to week 8. The F-GAN group received GAN feces, the F-G5P group received G5P feces, and the F-G5PH group received G5P feces after heat treatment (100°C, 10 min) and 0.22 μm sterile filtration.

**Experiment 3:** To study the health effects of ILA (Cat# I157602; Sigma Aldrich, USA), forty 6-week-old male mice were divided into four groups and fed a GAN diet. The groups received ILA solution intragastrically (in 0.5% CMC-Na) at 0, 10, 20, or 40 mg/kg every other day for 8 weeks. This dosage was based on previous studies 13,22. For the ILA suspension preparation, 40 mg of ILA was weighed out and ground with a small quantity of 0.5% CMC-Na solution. Thereafter, a constant volume of 0.5% CMC-Na solution was used to prepare a 10 mL suspension, yielding a concentration of 4 mg/mL. This suspension was then diluted to obtain 2 and 1 mg/mL suspensions.

**Experiment 4:** Thirty 6-week-old male C57BL/6J mice were randomly divided into three groups, G5P, G5P+Amp, and G5P+Amp+ILA, to further elucidate the role of the gut microbiota and the rescue effect of ILA. All groups were fed the G5P diet. The G5P+Amp group was administered 80 mg/kg BW ampicillin every other day, whereas the G5P+Amp+ILA group received 80 mg/kg BW ampicillin and 20 mg/kg BW ILA every other day.

At the end of the animal experiments, blood was collected and centrifuged at 4°C and 2500 rpm for 15 minutes to obtain serum samples. Organs were collected, immediately placed in liquid nitrogen, and stored at -80°C for further analysis. Fresh feces were collected in sterile tubes at week 8 and immediately stored at -80°C.

### 2.3. Oral glucose tolerance test (OGTT)

The animals fasted for 8 h before the glucose tolerance test. Blood glucose levels were determined at 0, 15, 30, 60, 90, and 120 min after oral gavage of glucose at 2 g/kg body weight with a GA-3 blood glucose meter (Sinocare Biology Co., China) 23.

### 2.4. Mouse body composition analysis

The small animal magnetic resonance imaging (MRI) system (Niumai, Suzhou, China) was calibrated with the provided standards and used to measure the body fat, water, and muscle masses of live mice.

### 2.5. Mouse liver histological analysis

The mouse livers were fixed in 4% paraformaldehyde for 24 h. For hematoxylin‒eosin (H&E) staining, liver samples were sectioned at a thickness of 5 μm after paraffin embedding. For Oil Red O (ORO) staining, fresh mouse livers were placed in optimal cutting temperature compound (OCT) and cut into 8 μm slices. The staining process was described in a previous study 24. The images were viewed under a light microscope (Nikon Eclipse E100, Nikon, Japan) at 400× magnification. ImageJ software (National Institutes of Health, USA) was used to measure the ORO-positive area in the liver tissue. H&E and ORO staining of mouse liver sections was used to calculate the NAFLD activity score (NAS) according to the American Association for the Study of Liver Diseases (AASLD) protocol 25.

### 2.6. Biochemical parameter detection

Serum biochemical parameters were detected using an automatic biochemical analyzer. The parameters included total triglycerides (TG, Cat#105-001396-00, Mindray, China), TC (Cat#105-000449-00, Mindray, China), high-density lipoprotein (HDL, Cat#105-000463-00, Mindray, China), low-density lipoprotein (LDL, Cat#105-001411-00, Mindray, China), alanine aminotransferase (ALT, Cat#105-000442-00, Mindray, China), and aspartate aminotransferase (AST, Cat#105-000443-00, Mindray, China). Mouse liver and feces were lysed using NP-40, and the supernatants were subsequently analyzed.

Tissue biochemical parameters, including TG, TC, and total bile acid (TBA, E003-2-1, Jiancheng, Nanjing), were detected with an automatic biochemical analyzer after homogenization, then and extracted in 75% ethanol at 50°C for 4 h, as previously described.

### 2.7. Reverse transcription and real-time PCR assay

Total RNA from mouse liver and HepG2 cells was extracted using SteadyPure RNA extraction kits (Cat#AG21023, Accurate Biology, China) and assayed for concentration and purity using NanoDrop. gDNA was removed and RNA was reverse-transcribed using the Evo M-MLV reverse transcription premix kit (Cat#AG11728, Accurate Biology, China). Real-time PCR was performed using one-step SYBR Green master mix (Cat#AG11719, Accurate Biology, China) on an Applied Biosystems QuantStudio 6 Pro instrument (Thermo Fisher, USA). The primer sequences are shown in Table S1. The expression levels were calculated using the 2^-ΔΔCt^ method and normalized to Actb 26.

### 2.8. 16S rRNA sequencing and analysis

Total DNA was extracted from feces using the Qiagen PowerSoil Pro Kit. The V3-V4 hypervariable region of the 16S rRNA gene was amplified using barcoded 341F/806R primers, and the products were analyzed with an Agilent 5400 Fragment Analyzer (Agilent, USA). The Deblur algorithm (v1.1.1) was used to denoise and generate amplicon sequence variants (ASVs). Taxonomic annotation was performed using Mothur software (v1.4.8) and the SILVA SSUrRNA database 27. The data were normalized for subsequent analysis.

Alpha diversity indices were calculated using the phyloseq (v1.4.0) and vegan (v2.6.2) R packages. Beta diversity analysis was conducted, and PCAs were generated. T-tests were used for comparisons between groups. LEfSe analysis was performed using LEfSe software (v1.1.2). PICRUSt2 (v2.5.0) was used for functional prediction. Intergroup differences were analyzed using t-tests.

### 2.9. Metabolomic profiling and analysis

MetWare Biotechnology Co., Ltd. (Wuhan, China) performed untargeted metabolomic analysis using an LC-ESI-MS/MS system (UPLC, ExionLC AD, MS, QTRAP). The analytical conditions were as follows: Waters C18 column (1.8 µm, 2.1 mm*100 mm); 40°C column temperature; 0.4 mL/min flow rate; 2 µL injection volume; water (0.1% formic acid):acetonitrile (0.1% formic acid) solvent system; gradient program: 95:5 V/V at 0 min, 10:90 V/V at 11.0 min, 10:90 V/V at 12.0 min, 95:5 V/V at 12.1 min, 95:5 V/V at 14.0 min. Principal component analysis (PCA) was performed using the prcomp function in R. Differential metabolites were determined according to the VIP (VIP > 1) and P value (P value < 0.05, Student's t test). VIP values were extracted from the OPLS-DA results, which also contain score plots and permutation plots, using the R package MetaboAnalystR 28. Metabolites were annotated using the KEGG database. Significantly regulated pathways were analyzed using metabolite set enrichment analysis (MSEA) 29.

### 2.10. ILA level detection

The concentrations of serum ILA were measured with a mouse ILA ELISA Kit (Cat#JL47423, Jianglai, Shanghai) according to the manufacturer's instructions. The ILA levels of the supernatants of in vitro bacterial cultures were also detected using an ELISA kit after tenfold concentration by nitrogen blowing at low temperature and re-solubilizing with dimethyl sulfoxide.

### 2.11. Molecular docking using AutoDock

First, the protein and ligand structures were downloaded from the protein data bank (PDB) and the PubChem database, respectively. Molecular docking and calculations were performed with AutoDock Vina (v1.5.7), and the results were visualized using PyMOL (v3.0.3) 30.

### 2.12. Total protein extraction and western blotting

Mouse liver or HepG2 cell samples were ground in lysis buffer supplemented with protease inhibitors and centrifuged to obtain the supernatant. The lysate containing 30 μg of protein was boiled, separated by 5-12.5% SDS-PAGE and blotted onto polyvinylidene fluoride membranes (0.45 μm, Bio-Rad). After blocking with 7% milk, the blots were incubated with anti-GAPDH (CST, 2118, 1:1000), anti-FXR/NR1H4 (CST, 72105, 1:1000), anti-CYP7A1 (Abcam, ab65596, 1:1000), anti-ABCB11/BSEP (Abcam, ab315474, 1:1000), and anti-ABCG8 (Abcam, ab223056, 1:2000) antibodies, followed by incubation with HRP-conjugated horse anti-mouse IgG (CST, 7076, 1:2000) and goat anti-rabbit IgG (CST, 7074, 1:2000) where appropriate 31.

### 2.13. Cell culture and treatment

HepG2 In Vitro High-Fat Cell Model Construction: A high-fat model of HepG2 cells was established by treating the cells with complete medium containing 600 μM free fatty acids (FFA) (400 μM oleic acid (OA) + 200 μM palmitic acid (PA)) for 24 h 32.

Investigation of Intestinal Metabolites and Heat Treatment Effects: Fecal slurry suspensions from G5P mice were diluted 2400, 1200, 600, 300, and 150 times with complete medium containing 600 μM FFA. These dilutions were used to treat HepG2 cells for 24 hours to explore the effects of intestinal metabolites and heat treatment.

Exploration of the In Vitro Effects of ILA: Solutions at concentrations of 250, 500, and 1000 μM were prepared and used to treat HepG2 cells for 24 hours.

Verification of FXR as an Upstream Regulatory Molecule: Small interfering RNA (siRNA, Ruibo, Guangzhou, China) targeting FXR and the lipid transfection reagent Lipo3000 (Thermo Fisher, Cat#L3000-015, USA) were diluted separately in reduced-serum medium (Opti-MEM, GIBCO, Cat#31985070, USA). The two solutions were mixed at a 1:1 ratio and incubated at room temperature in the dark for 15 min. The transfection complex was evenly distributed into the wells to achieve a final siRNA concentration of 50 nM. The cells were incubated for 6 hours, after which the medium was replaced. Following washing with PBS, the cells were treated with 500 μM ILA or switched to complete medium. The protein and mRNA levels were measured within 72 hours.

### 2.14. *In vitro* bacterial culture of *A. muciniphila* and *E. coli* with or without pectin

*A. muciniphila* (ATCC BAA-835) and *E. coli* (ATCC 25922) were cultured for use in this study. The strains were cultivated anaerobically at 37°C in brain heart infusion (BHI) medium supplemented with 0.5% type II mucin, 0.05% L-cysteine, and 0.14 g/L tryptophan for 48 h. Fermented samples were immediately centrifuged at 12500 rpm for 5 min. The supernatants were filtered through a 0.22 μm filter and then frozen at -20°C for subsequent quantification of ILA.

### 2.15. Statistical analysis

Data analysis was conducted using SPSS (version 22), and GraphPad Prism (version 9.0.2) was used for data visualization and graph creation. Group differences were assessed using unpaired t-tests or one-way ANOVA after both normality checks with Shapiro-Wilk tests and variance homogeneity checks with Levene's test. For multifactor comparisons, two-way ANOVA combined with post-hoc Bonferroni correction was used. The results are expressed as the mean ± standard deviation, with statistical significance set at p < 0.05. Symbols denote significance levels: * or # for *p* < 0.05, ** or ## for *p* < 0.01, *** or ### for *p* < 0.001, **** or #### for *p* < 0.0001.

## 3. Results

### 3.1. Molecular weight and FT-IR analysis of pectin

The molecular weight of the citrus pectin used in this study was analyzed by GPC. The chromatograms (Figure S1A) revealed a prominent broad peak (Peak 1) and a weak peak. The main peak had a molecular weight (Mp) of 85.6 kDa, a number average molecular weight (Mn) of 32.4 kDa, a weight average molecular weight (Mw) of 150.4 kDa, and an area of 86.71% (Figure S1A).

The pectin from the three production batches used in this study was characterized by FTIR (Figure S1B), which revealed saccharide absorption peaks. Based on absorption peaks at 1730 cm⁻¹ and 1630 cm⁻¹, the degree of esterification (DE) was 59.01% (calculated by A₁₇₃₀ / (A₁₇₃₀+A₁₆₃₀)). In addition, the purity of the pectin used in this study was characterized by its galacturonic acid content, which was determined to be 78.12%.

### 3.2. Pectin ameliorated GAN diet-induced hepatic lipid accumulation and glycolipid metabolism disorders by enhancing bile acid synthesis and excretion

**Figure 1:**
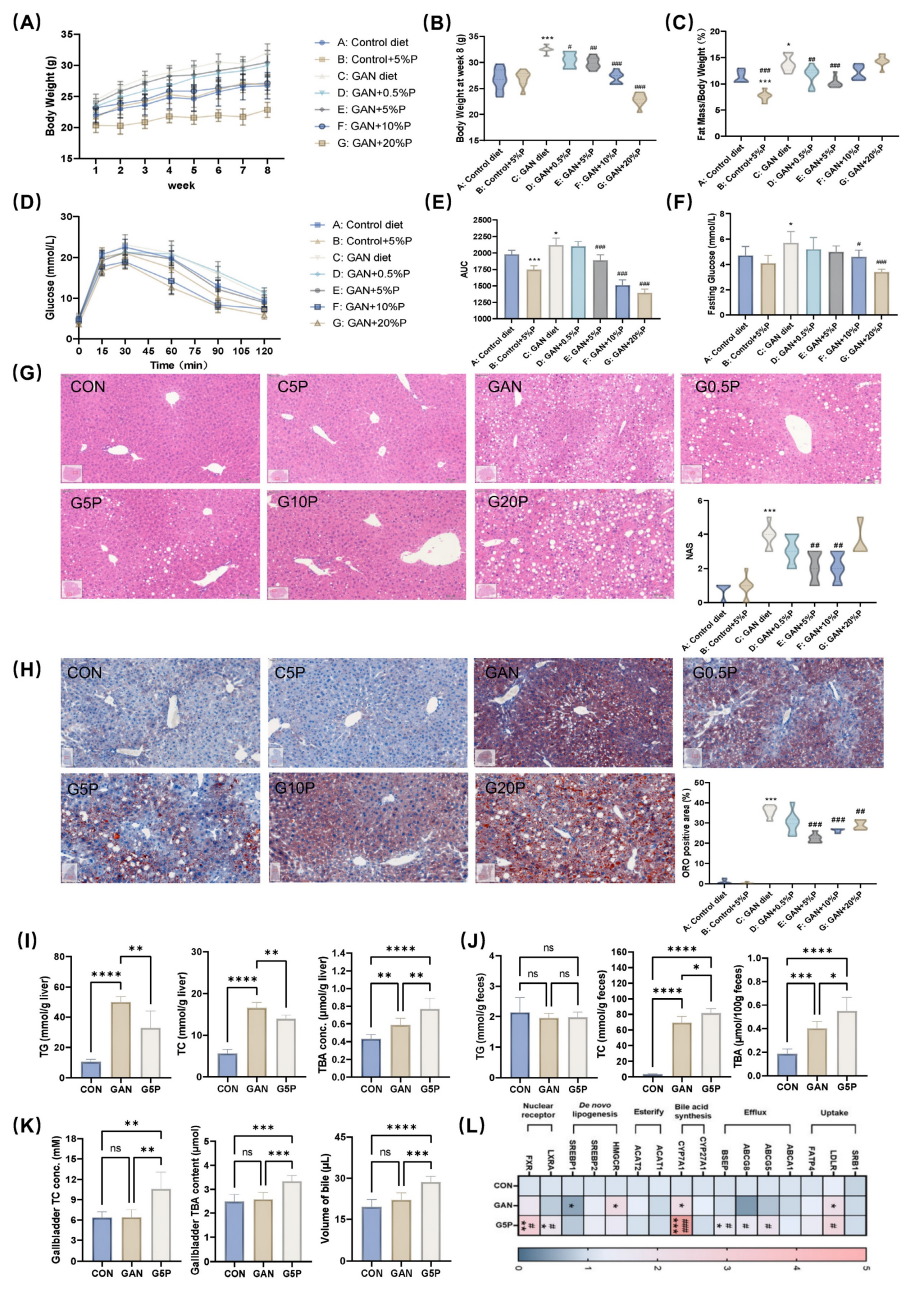
Pectin ameliorated GAN-induced hepatic lipid accumulation and glycolipid metabolism disorders related to enhancing bile acid synthesis and excretion. (A) Mouse body weight during 8 weeks of pectin intervention; (B) Mouse body weight at week 8; (C) Mouse fat mass ratio at week 8; (D) Glucose levels during OGTT; (E) Area under OGTT curve; (F) Fasting glucose level; (G) Representative images of H&E stained mouse liver sections and NAS; (H) Representative images of ORO stained mouse liver sections and their positive area quantification; (I) Mouse liver TG, TC, and TBA levels; (J) Mouse feces TG, TC, and TBA levels; (K) Gallbladder TC and TBA levels and volume of gallbladder bile; (L) Mouse liver mRNA levels of bile acid- and cholesterol metabolism-related genes. * indicates a significant difference between GAN and CON; # indicates a significant difference between G5P and GAN.

To investigate the health effects of pectin dietary intervention on GAN diet-induced MAFLD, the mice were supplemented with varying doses of pectin for 8 weeks (Figure S2A). During the 8-week intervention period, food intake among the groups remained similar (Figure S2B), except for the G20P group. The results demonstrated that the addition of 5% and 10% pectin to the GAN diet significantly reduced body weight and body fat percentage and improved glucose tolerance in the mice (Figure 1A-F). Furthermore, the addition of 5% pectin to the GAN diet significantly improved the blood lipid profile in mice (Figure S2C). Histological analysis of liver sections using H&E, NAS, and ORO staining, along with quantitative assessment of positive areas, revealed significant alleviation of hepatic pathology and reduced lipid accumulation by 5% and 10% pectin (Figure 1G and H). These results suggest that 5% pectin significantly improved glucose and lipid metabolism in mice fed a GAN diet.

Additionally, 5% pectin intervention significantly reduced hepatic TG and TC levels while increasing TC levels in feces and gallbladder bile (Figure 1I and J), suggesting that pectin mitigated lipid accumulation by increasing hepatic cholesterol excretion. Pectin treatment led to notable increases in TBA in the gallbladder, feces, and liver of pectin-treated mice (Figure 1I-K), indicating increased bile acid hepatic synthesis and excretion. Analysis of the mRNA levels of genes involved in hepatic cholesterol and bile acid metabolism revealed that pectin intervention significantly upregulated CYP7A1, BSEP, ABCG8, FXR, and LXRα expression (Figure 1L).

### 3.3. Pectin reshaped the gut microbiota composition, particularly increasing the abundance of *A. muciniphila* and *E. coli*

**Figure 2:**
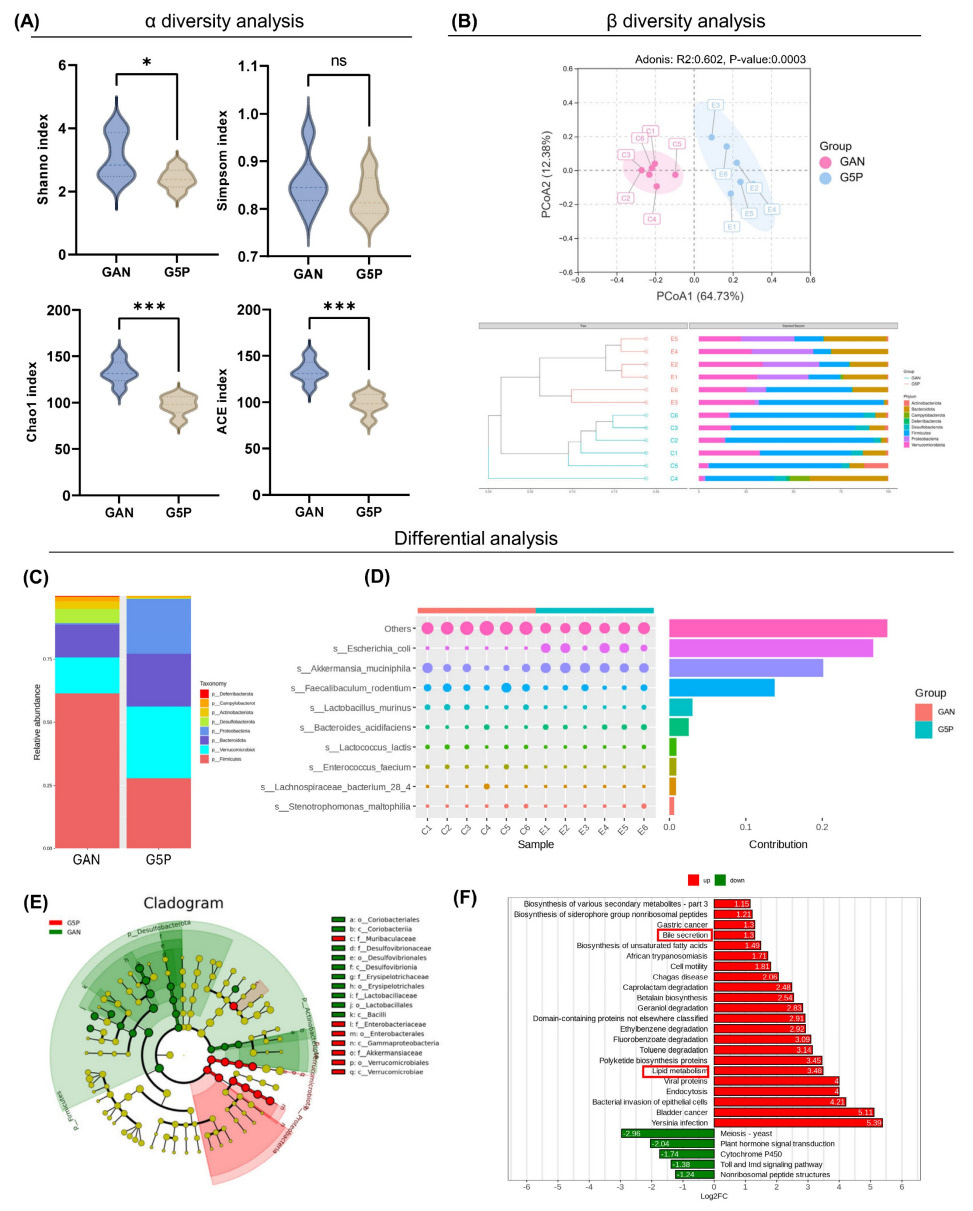
Pectin intervention reshaped gut microbiota composition, particularly increasing the abundance of *A. muciniphila* and* E. coli.* (A) α diversity analysis including Shanno, Simpson, Chao, and ACE index analysis; (B) β diversity analysis including PCoA and UPGMA analysis at phylum level; (C) Visualized ASV abundance at phylum level between groups; (D) The top ten contributing species and their abundance to the divergence between the two groups; (E) ASV-based evolutionary branching map, the circle radiating from inside to outside represents the phylum to genus level; (F) Bar graph of ASV-based differential pathways in PICRUSt2.

Consequently, 16S rRNA sequencing was used to investigate its regulatory impact on the gut microbiota. Compared with the GAN group, the 5% pectin intervention group presented significantly lower Shannon, Chao, and ACE indices, while no significant difference was observed in the Simpson index between the two groups (Figure 2A). These findings suggested that pectin intervention did not substantially increase intestinal microbial richness or diversity in mice. PCA based on ASV revealed a significant difference in species composition between the two groups (ANOSIM: R=0.813, P=0.003) (Figure 2B).

The disparity in the gut microbiota composition between the two groups was then investigated. Compared with the GAN group, the 5% pectin supplementation group presented a substantial increase in the relative abundance of *Bacteroidetes*, *Verrucomicrobia*, and *Proteobacteria*, which was concurrent with a decrease in the relative abundance of *Firmicutes* at the phylum level (Figure 2C). Our results demonstrated that pectin intervention led to a substantial decrease in the Firmicutes/Bacteroidetes (F/B) ratio, which may be indicative of its health-promoting effects.

To identify the microorganisms causing differences between the two groups, we used Simpson analysis to decompose the Bray-Curtis index and quantify the species contributions, as shown in Figure 2D (top 10). The top three contributors were the unrecognized species, *A. muciniphila,* and* E. coli*, whose abundances increased in the G5P group. Metabolic pathway enrichment analysis was subsequently conducted, with the results illustrated in Figure 2F. The analysis revealed that the alteration of the gut microbial flora resulting from pectin intervention enhanced lipid metabolism and bile excretion capacity. The results indicated that pectin enhanced glycolipid metabolism by modifying the composition of the gut flora, a phenomenon potentially associated with increased levels of *A. muciniphila* and* E. coli.*

### 3.4. Pectin treatment regulated bile acid synthesis and excretion through the gut microbiota and its metabolites

**Figure 3:**
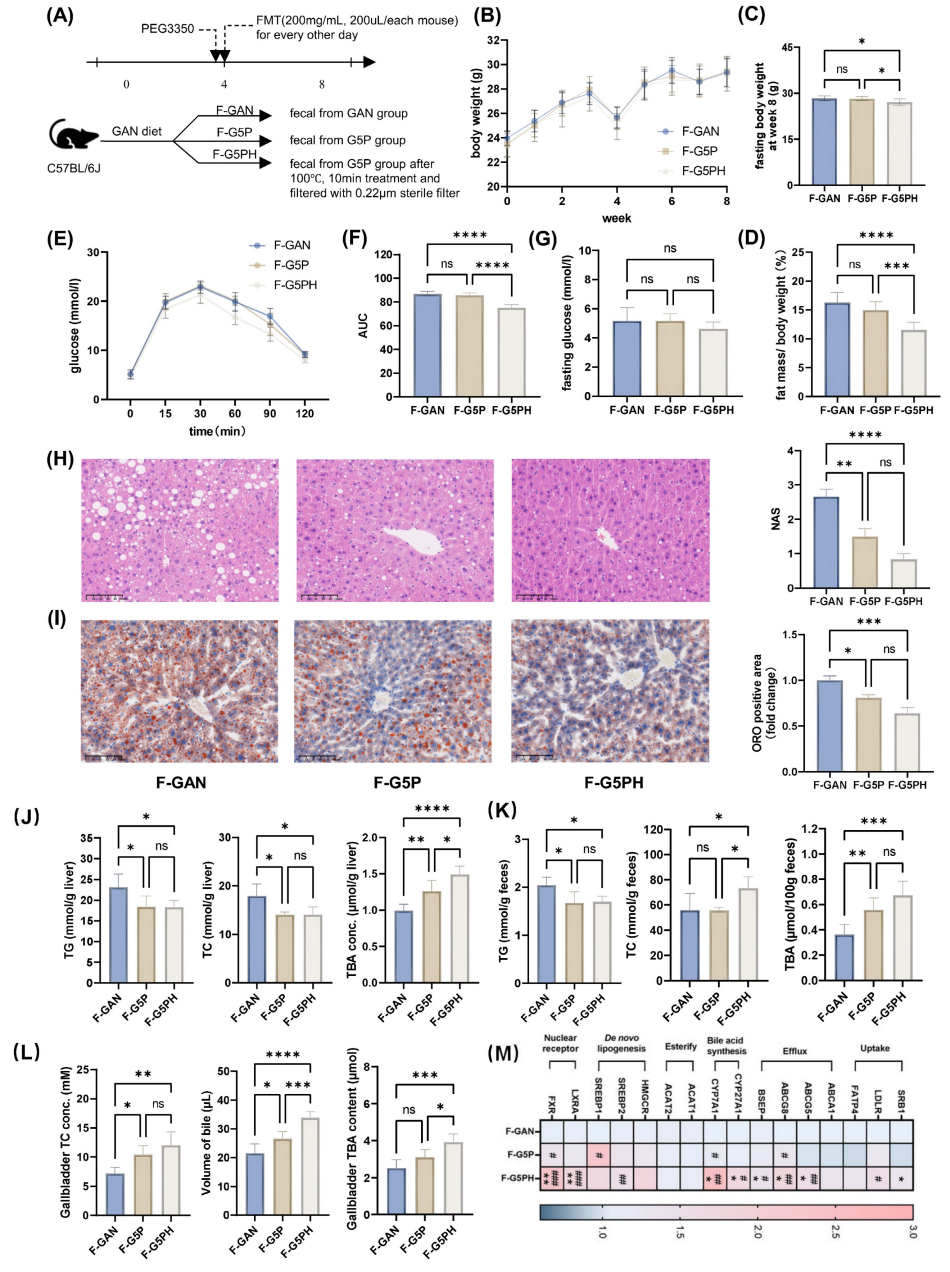
Gut microbiota and their metabolites mediate the regulation of bile acid synthesis and excretion by pectin, thereby improving hepatic lipid accumulation. (A) Schematic design of the animal experiment; (B) Mouse body weight during experiment; (C) Mouse body weight at week 8; (D) Mouse fat mass ratio at week 8; (E) Glucose levels during OGTT; (F) Area under OGTT curve; (G) Fasting glucose level; (H) Representative images of H&E stained mouse liver sections and NAS; (I) Representative images of ORO stained mouse liver sections and their positive quantification; (J) Mouse liver TG, TC, and TBA levels; (K) Mouse feces TG, TC, and TBA levels; (L) Gallbladder TC and TBA levels and volume of gallbladder bile; (M) Mouse liver mRNA levels of bile acid metabolism- and cholesterol-related genes. * indicates significant difference between F-G5P and F-GAN; # indicates significant difference between F-G5PH and F-G5P.

To study the role of the gut microbiota and its metabolites after pectin intervention, we conducted FMT experiments on mice with fecal slurry suspensions made from feces of GAN (F-GAN), G5P (F-G5P), and heat-treated (100°C for 10 min, 0.22 μm sterile filtered) G5P fecal slurry suspensions (F-G5PH) (Figure 3A).

FMT experiments revealed no significant differences in food intake among the groups (Figures 3B and S2D). There was no significant improvement in body weight or body fat percentage in the F-G5P group, whereas a significant reduction was observed in the F-G5PH group (Figure 3C and D).

The fasting blood glucose levels among the three groups were comparable (Figure 3G). Nevertheless, F-G5PH treatment significantly improved glucose tolerance (Figure 3E and F). Both FMT with F-G5P and FMT with F-G5PH improved the mouse serum lipid profile (Figure S2E). Pathology of liver sections and detection of hepatic TG and TC levels clearly revealed that both F-G5P and F-G5PH administration significantly reduced hepatic lipid accumulation (Figure 3H-J). TC levels were increased in F-G5PH feces and gallbladders (Figure 3K and L). The TBA content was increased in the liver, gallbladder, and feces of mice in the F-G5P and F-G5PH groups (Figure 3J-L). Consistent with the results of the previous animal experiment, the mRNA levels of CYP7A1, BSEP, and ABCG8 in the liver were also significantly different between the F-G5PH and GAN groups (Figure 3M).

### 3.5. Gut microbiota metabolites activate CYP7A1 and FXR-BSEP, which promote hepatic bile acid synthesis and excretion

**Figure 4:**
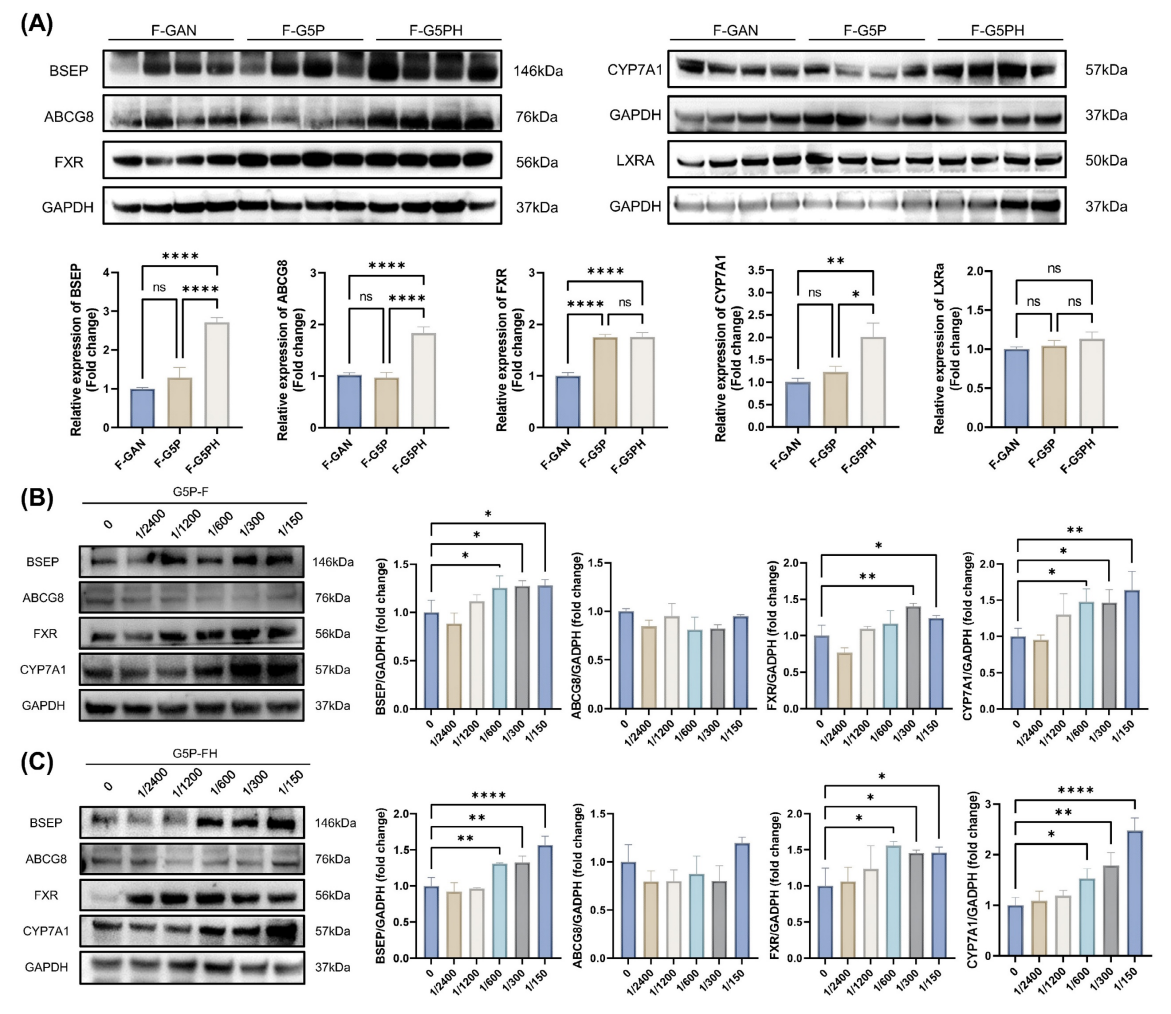
Gut microbiota metabolites activate CYP7A1 and FXR-BSEP to promote hepatic bile acid synthesis and excretion. (A) Cholesterol and bile acid metabolism-related protein levels in mouse liver; (B)-(C) Related protein levels in HepG2 cells after feces slurry intervention with or without heat treatment, which were generated from fecal samples from G5P mice.

To further validate the molecular mechanisms underlying the health effects of intestinal metabolites, the expression levels of relevant proteins in the livers of mice from animal experiment 2 were examined (Figure 4A). Concurrently, an in vitro high-fat model using HepG2 cells was established, and the cells were treated with sterile fecal slurry suspensions from pectin-treated mice for 24 h, followed by assessment of related protein levels (Figure 4B and C). The results revealed that, both in vivo and in vitro, gut microbiota metabolites from pectin supplementation mediated the activation of hepatic CYP7A1 and FXR-BSEP.

### 3.6. Pectin intervention increased tryptophan-derived ILA by the gut microbiota, which was associated mainly with the abundance of *A. muciniphila* and *E. coli*

**Figure 5:**
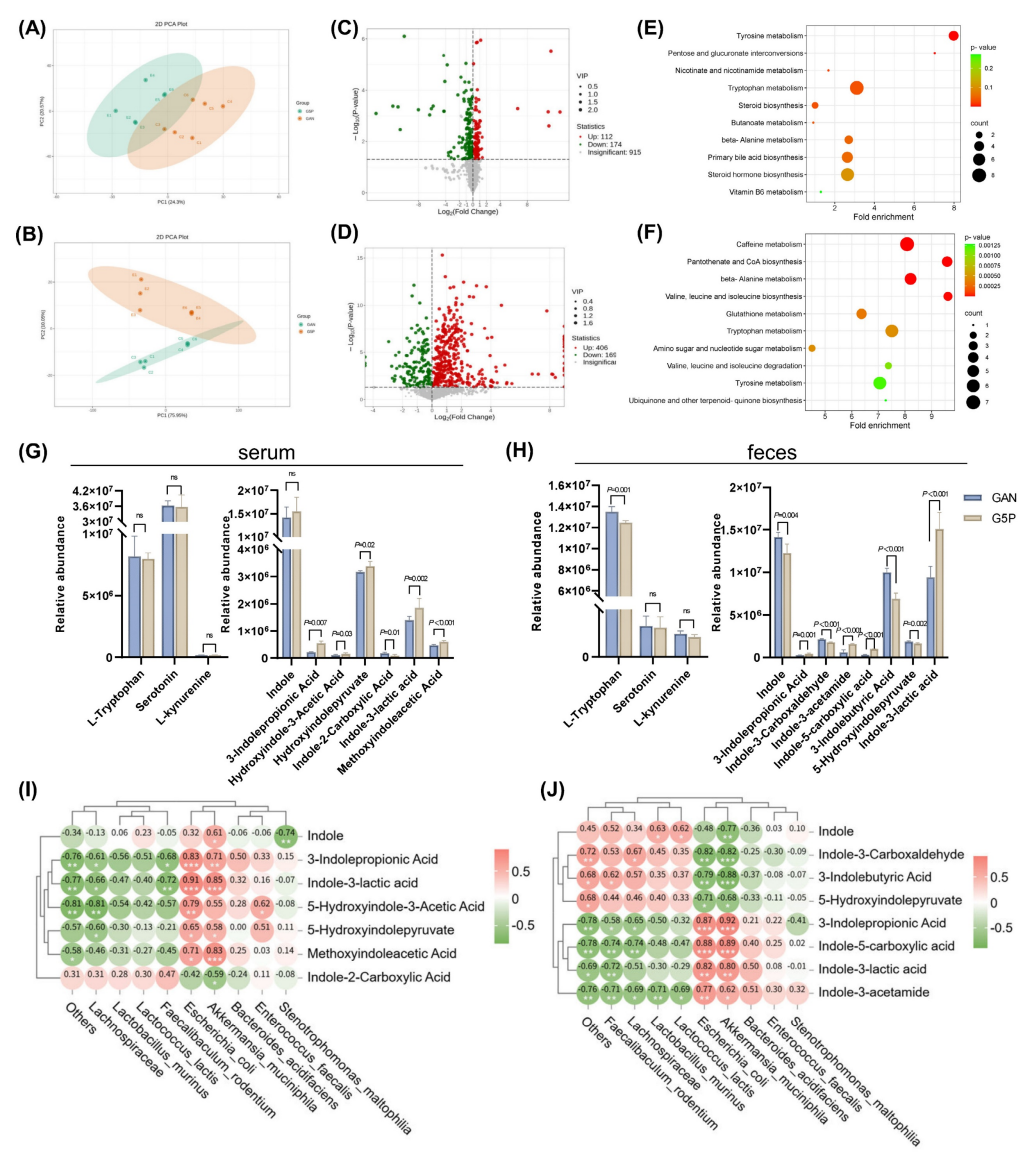
Pectin intervention regulated intestinal tryptophan metabolism via increased ILA levels in feces and serum of mice, which was positively correlated with *A. muciniphila* and *E. coli* abundance. (A) Serum metabolites PCA plot; (B) Feces metabolites PCA plot; (C) Volcano plot of differential metabolites in the serum; (D) Volcano plot of differential metabolites in the feces; (E) Serum differential metabolites MSEA enrichment analysis; (F) Feces differential metabolites MSEA enrichment analysis; (G) Mouse serum tryptophan metabolites relative abundance; (H) Mouse feces tryptophan metabolites relative abundance; (I) Spearman correlation analysis between mouse serum indole derivatives and the top ten differential contributors at genus level to the microbiota; (J) Spearman correlation analysis between mouse feces indole derivatives and the top ten differential contributors at species level to the microbiota.

We also utilized untargeted metabolomics technology to analyze metabolites in mouse serum and feces, further investigating the effects of pectin intervention. PCA revealed significant differences in the serum and fecal metabolite profiles between the GAN and G5P groups (Figure 5A and B).

Differential analysis revealed that, compared with those in the GAN group, 112 metabolites were significantly upregulated and 174 were downregulated in the serum of the G5P group, whereas 406 metabolites were significantly upregulated and 169 were downregulated in the feces of the G5P group (Figure 5C and D). We subsequently performed MSEA on the differential metabolites, revealing significant enrichment in the tryptophan metabolism pathway, which is closely associated with the gut flora (Figure 5E and F). The relative abundances of tryptophan metabolites in mouse serum and feces are shown in Figure 5G and H. For the kynurenine and serotonin pathways, no significant differences were observed in the serum levels of tryptophan, serotonin, or kynurenine. However, pectin intervention significantly reduced fecal tryptophan levels without affecting serotonin or kynurenine levels (Figure 5G and H). With respect to the indole pathway, serum indole levels did not significantly change, whereas the fecal indole levels significantly decreased after pectin intervention (Figure 5G and H). Differential analysis revealed significant differences in the levels of indoles and their derivatives between the two groups, as shown in Figure 5G and H. Among these, only indole-3-propionic acid (IPA) and ILA were significantly upregulated in both the serum and feces. Spearman correlation analysis was used to explore the relationships between significantly altered indole metabolites and the gut microbiota, revealing that ILA and IPA were significantly positively correlated with *A. muciniphila* and *E. coli* (Figure 5I and J).

To confirm the specific role of these strains in ILA generation, we also conducted in vitro fermentation experiments in which the strains were inoculated with *A. muciniphila* or *E. coli* with or without pectin supplementation. The results demonstrated that both *A. muciniphila* and *E. coli* culture markedly increased the ILA levels of the fermentation supernatants by approximately 2.2-fold and 1.8-fold, respectively (Figure S2F). Additional pectin supplementation could also significantly increase ILA levels in response to *A. muciniphila* or *E. coli* inoculation (Figure S2F).

### 3.7. ILA activated cell CYP7A1 and FXR-BSEP in a high-fat HepG2 model

**Figure 6:**
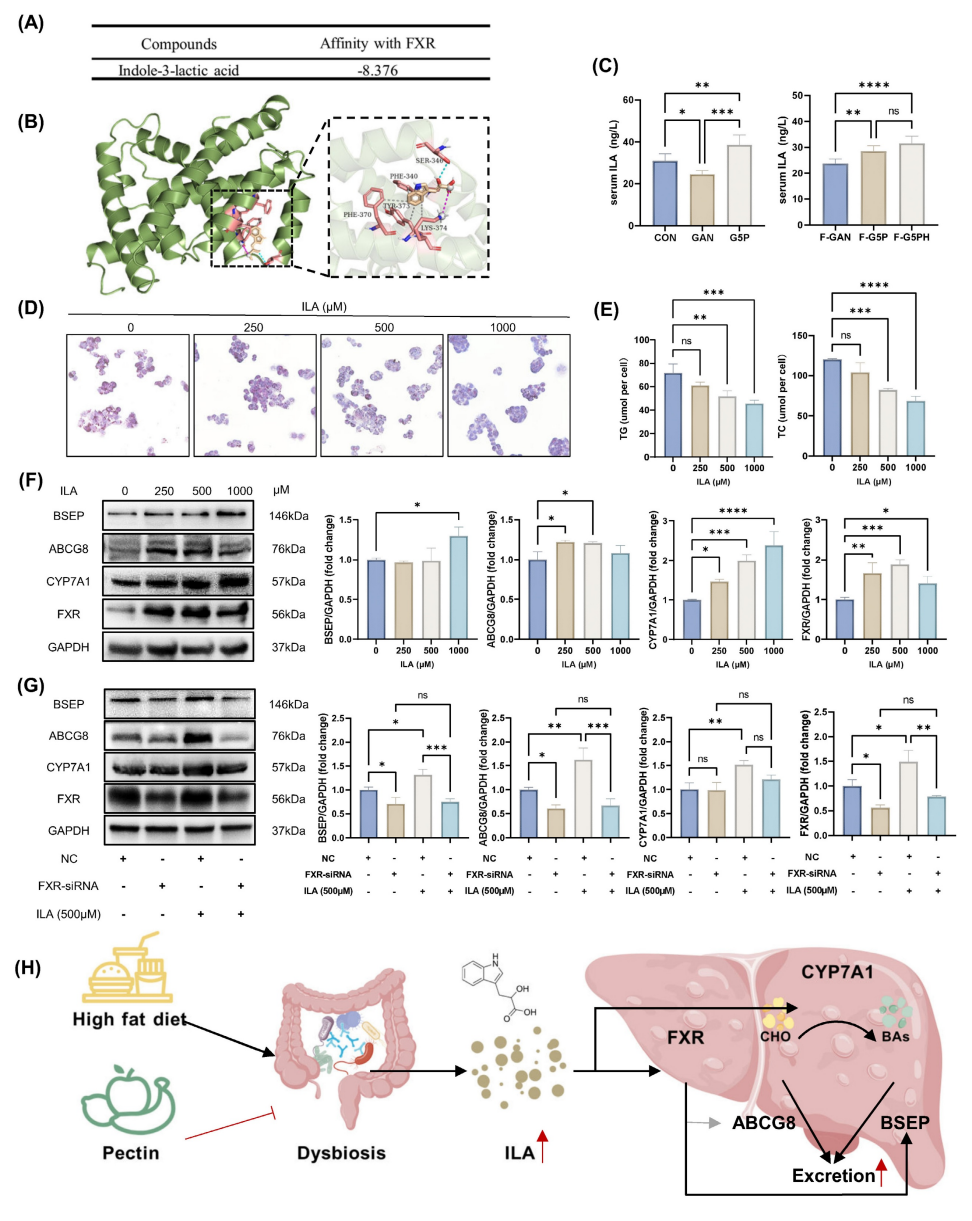
ILA activated HepG2 cell CYP7A1 and FXR-BSEP in vitro. (A) Molecular docking calculation of the affinity results of ILA with FXR; (B) Schematic diagram of the molecular docking mode of ILA and FXR; (C) Mouse serum ILA levels; (D)-(E) ORO stained HepG2 cell and cellular TG and TC levels with ILA treatment; (F) ILA treatment activated CYP7A1 and FXR-BSEP; (G) Effect of FXR knockdown on BSEP, ABCG8, and CYP7A1 protein expression levels; (H) Schematic of the molecular mechanism.

Compared with IPA, ILA exhibited greater abundance and more significant differences in both the serum and feces (Figure 5G and H). Using ELISA, we confirmed that both pectin intervention and the oral administration of a fecal slurry from pectin-treated mice elevated the serum ILA levels in mice (Figure 6C). Previous studies have reported that FXR, a key nuclear transcription factor in the liver, regulates the expression of BSEP and CYP7A1 3,33. We hypothesized that ILA, derived from the gut microbiota, acts on hepatic FXR via absorption by the intestinal epithelium and subsequent entry into the circulation. Therefore, molecular docking simulations were used to explore the binding mode of ILA to FXR. The results showed that ILA could form a hydrogen bond at Ser-346; hydrophobic interactions at PHE-340, TYR-373, and LYS-374; and a salt bridge at LYS-374 with FXR (Figure 6A and B).

In an in vitro high-fat HepG2 cell model, ILA intervention at various doses (250-1000 μM) significantly reduced intracellular lipid accumulation, as evidenced by decreased ORO-positive areas and reduced TG and TC levels (Figure 6D and E). ILA intervention also effectively activated CYP7A1 and FXR-BSEP protein expression (Figure 6F). To further elucidate the upstream regulatory role of FXR, we used siRNA to suppress FXR expression in the high-fat HepG2 model, followed by ILA intervention and assessment of related protein levels (Figure 6G). In the negative control group, ILA intervention significantly increased the expression levels of BSEP, ABCG8, CYP7A1, and FXR (Figure 6G). Under FXR suppression by siRNA, ILA intervention failed to increase the expression of BSEP and ABCG8 but did not significantly affect CYP7A1 expression (Figure 6G). This phenomenon suggested that ILA may stimulate CYP7A1 expression by other regulatory pathways in addition to FXR, although further investigation is needed. We hypothesized that ILA-induced CYP7A1 upregulation was partially independent of FXR regulation. The molecular mechanism is shown in Figure 6H.

### 3.8. ILA intragastric administration improved liver lipid accumulation in mice by activating CYP7A1 and FXR-BSEP

**Figure 7:**
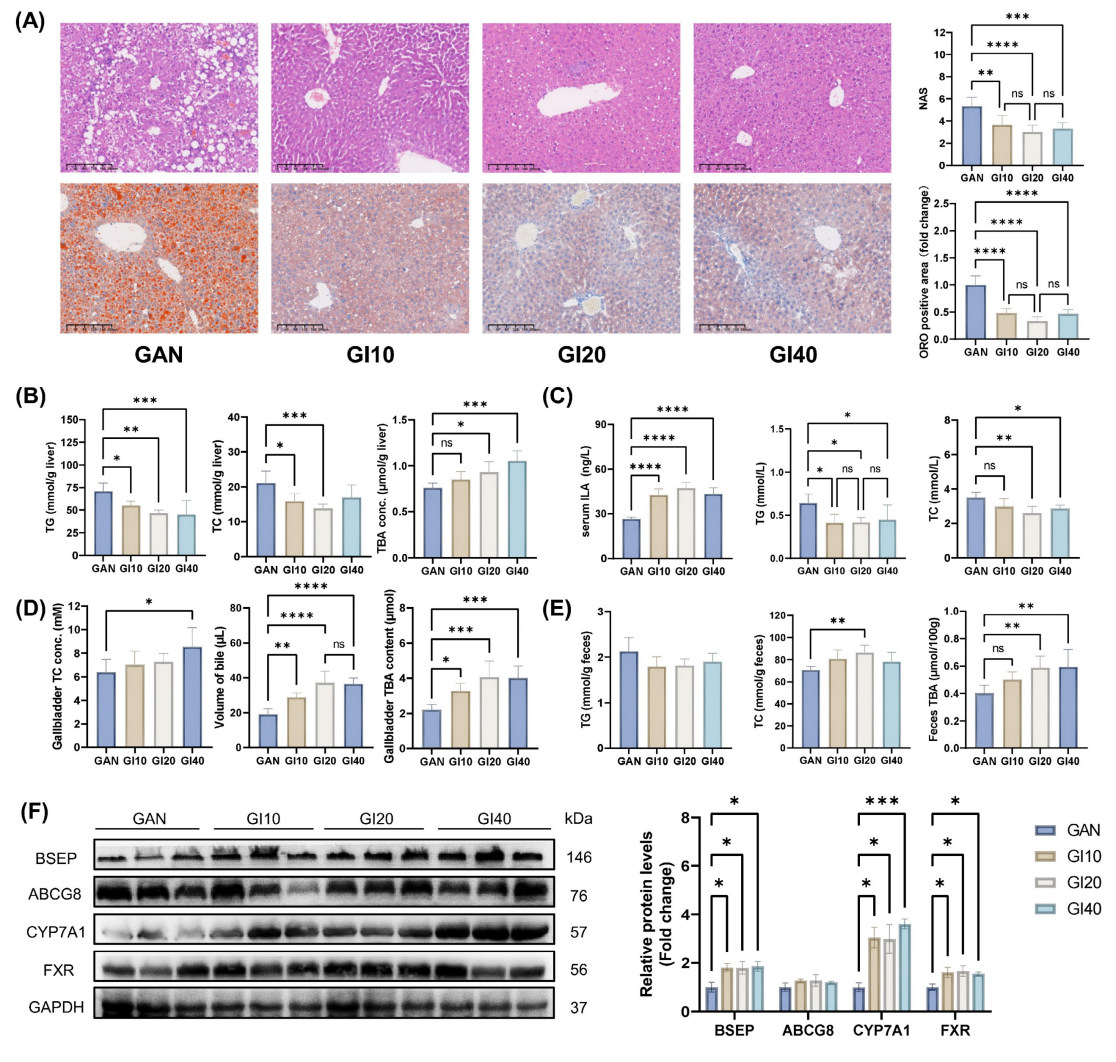
Different doses of intragastric ILA administration improved liver lipid accumulation in mice by activating CYP7A1 and FXR-BSEP. (A) Representative images of H&E and ORO-stained mouse liver sections, NAS levels, and ORO-positive area quantification; (B) Mouse liver TG, TC, and TBA levels; (C) Mouse serum ILA, TG, and TC levels; (D) Gallbladder TC and TBA levels and volume of gallbladder bile; (E) Mouse feces TG, TC, and TBA levels; (F) Related protein levels in mouse liver.

We further investigated the effects of intragastric ILA administration at different doses on MAFLD in mice. All ILA doses (10-40 mg/kg BW) improved liver pathology and reduced lipid accumulation in mice, as evidenced by decreased liver NAS, ORO-positive area (Figure 7A), and hepatic TG and TC levels (Figure 7B). After 8 weeks of intervention with ILA, the serum ILA levels increased significantly (Figure 7C). The serum TG, TC, ALT, and AST levels also decreased significantly (Figures 7C and S3E). TBA levels in the liver, gallbladder, and feces markedly increased with ILA treatment (Figure 7D and E). Compared with those in the GAN group, the fecal TC levels were significantly greater in the GI20 group (Figure 7E). Western blot analysis revealed that ILA intervention significantly upregulated the expression of CYP7A1, BSEP, and FXR (Figure 7F). The expression of ABCG8 tended to increase but was not significantly different.

These results suggest that 10 and 20 mg/kg BW ILA ameliorates diet-induced liver lipid accumulation, dyslipidemia, and liver injury and enhances hepatic bile acid synthesis and excretion by activating CYP7A1 and FXR-BSEP. However, 40 mg/kg BW ILA may cause mild liver damage.

### 3.9. ILA supplementation alleviated MAFLD caused by antibiotic-induced dysbiosis of the gut microbiota

To further confirm that the augmented ILA in mouse serum after pectin intervention originated from the gut microbiota and that ILA supplementation could counteract the adverse impacts of gut dysbiosis, we performed a rescue experiment by supplementing ILA while inducing gut microbiota dysbiosis with antibiotics.

**Figure 8:**
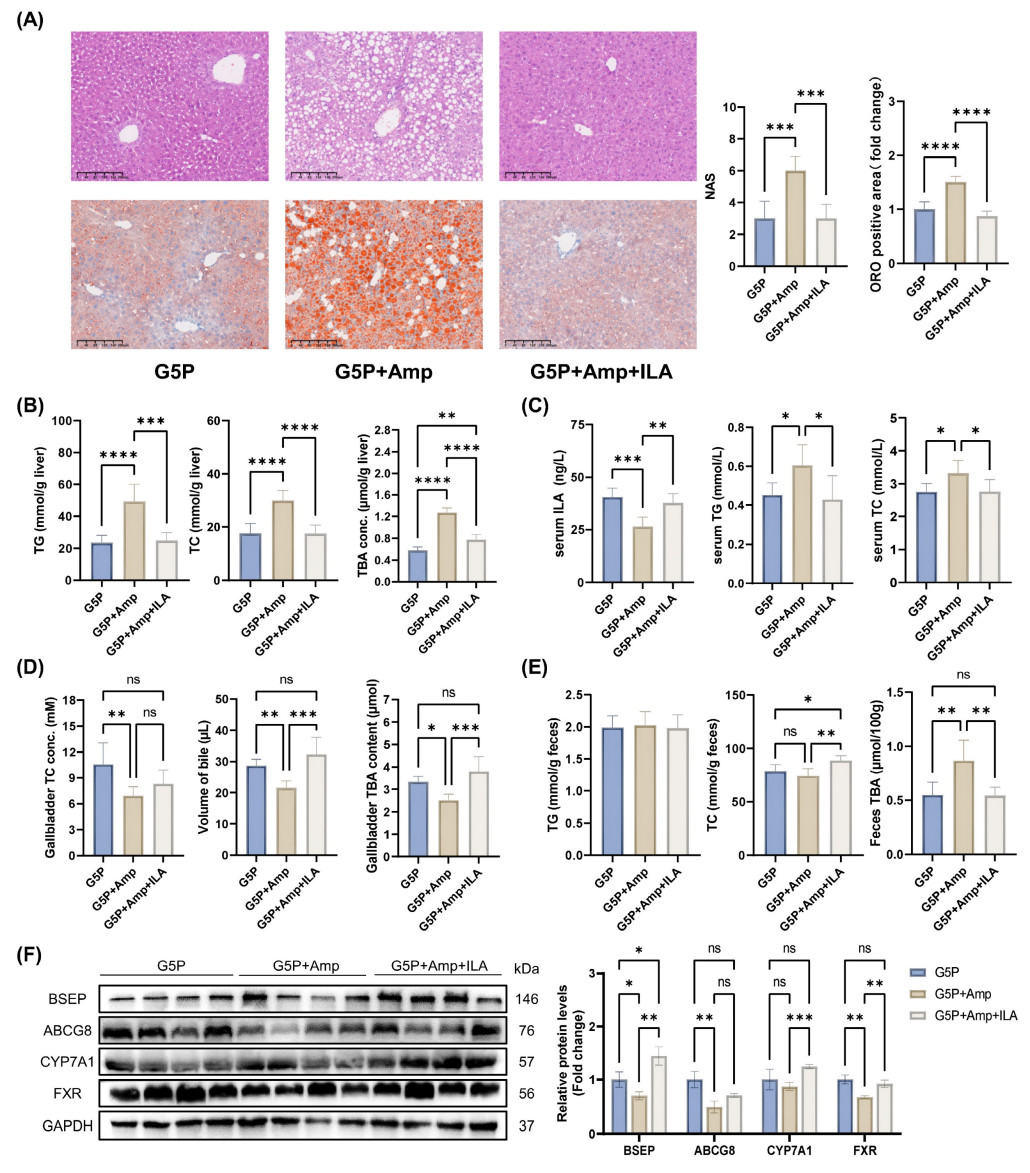
Serum ILA level is regulated by intestinal flora, and ILA supplementation can alleviate the adverse effects caused by dysbiosis. (A) Representative images of H&E and ORO-stained mouse liver sections, NAS levels, and ORO-positive area quantification; (B) Mouse liver TG, TC, and TBA levels; (C) Mouse serum ILA, TG, and TC levels; (D) Gallbladder TC and TBA levels and volume of gallbladder bile; (E) Mouse feces TG, TC, and TBA; (F) Related protein levels in mouse liver.

The administration of antibiotics for 8 weeks resulted in substantial increases in the liver NAS, ORO-positive area, and hepatic and serum TG and TC levels (Figure 8A-C). In addition, the treatment led to a notable increase in the serum ALT and AST levels, as shown in Figure S3J. A G5P diet and antibiotic-induced gut dysbiosis significantly reduced serum ILA levels in mice (Figure 8C). However, intragastric administration of ILA restored the serum ILA concentration to a level comparable to that in the G5P group (Figure 8C). Additionally, gut dysbiosis reduced gallbladder bile acid content, volume, and TC levels, which were restored to those of the G5P group by ILA supplementation (Figure 8D and E). In feces, intestinal flora disorders caused by ampicillin had no significant effect on the TG and TC levels but significantly increased the TBA levels (Figure 8E). In the context of intestinal flora dysbiosis, ILA intervention effectively increased fecal TC levels and restored liver and fecal TBA levels to normal (Figure 8E). Moreover, ILA could reactivate CYP7A1 and FXR-BSEP under antibiotic intervention (Figure 8F).

## 4. Discussion

Despite the well-documented benefits of dietary fiber on lipid metabolism and hepatic steatosis, the underlying mechanisms, particularly the role of microbiota-derived metabolites, remain poorly understood. The present study integrated gut microbial analysis and metabolomic profiling to elucidate a novel mechanism by which pectin intervention impacts gut microbiota metabolism and liver steatosis.

Fatty liver disease has emerged as a critical health issue in humans. Experimental evidence has demonstrated that consumption of a GAN diet can induce MAFLD in murine models, which is characterized by hepatic steatosis and the accumulation of fatty acids and cholesterol within the liver tissue 34. Consequently, the GAN diet has been used to establish animal models of MAFLD. In the present study, C57BL/6J mice fed a GAN diet for 8 continuous weeks exhibited features of MAFLD. The results also revealed increased cholesterol and triglyceride accumulation in hepatocytes and serum, which could be decreased by pectin supplementation. In addition, the implementation of a 5% pectin intervention resulted in substantial increases in bile acid levels within the liver, feces, and gallbladder. This observation indicates that pectin enhances bile acid synthesis and subsequent excretion from the liver.

Interestingly, the G20P group demonstrated an absence of a protective effect against MAFLD in this study. At autopsy, among all the dose groups, only one-third of the mice in the G20P group developed intestinal distension. It was hypothesized that excessive pectin fermentation by intestinal bacteria would result in greater gas byproduct generation, leading to intestinal distension and subsequent fullness, which would affect appetite and psychology. Additionally, fermentable fibers can lead to faster depletion of macronutrients and micronutrients in mice 35. Consequently, inadequate caloric intake and increased nutrient clearance due to the high proportion of pectin in the diet may have contributed to the nonprotective effect of MAFLD in the G20P group.

The interaction between dietary fiber and the gut microbiota strongly influences host metabolic health. Dietary pectin has been demonstrated to potentially influence the composition of the gastrointestinal microbiota and gut metabolites, which may affect the metabolic homeostasis of the host 36,37. It has been proposed that dietary pectin may reverse gut microbial dysbiosis caused by a high-fat diet 37. As documented in earlier studies of prebiotic effects, adding citrus pectin to the diet changed the gut microbiota composition, characterized by an increased abundance of *Bacteroidetes* and a reduced abundance of *Firmicutes* 38. While our findings corroborate the general consensus that pectin reduces the F/B ratio, a marker of dysbiosis in metabolic disorders, the genus-level shifts observed in this study diverge from earlier reports. For example, apple pectin has been demonstrated to enrich *Lactobacillus* and *Bifidobacterium* 39, whereas citrus pectin has been observed to preferentially increase *Enterobacteriaceae* and suppress *Faecalibaculum*. These discrepancies are likely attributable to variations in the physicochemical properties and experimental conditions of pectin. The high galacturonic acid content and moderate esterification (59.01%) of the citrus pectin used in this study may favor colonization by species capable of degrading methyl-esterified homogalacturonan.

At the species level, pectin intervention resulted in the enrichment of *A. muciniphila* and *E. coli*, which was also observed in previous studies 40. *A. muciniphila* is a mucin-degrading bacterium associated with improved metabolic health. Its increased abundance has been shown to contribute to antiobesity effects, improving metabolic disorders and MAFLD 41. In addition, recent studies have confirmed that both* A. muciniphila* and *E. coli* are able to produce ILA 19,42, establishing a positive link between gut bacteria and increased ILA.

A series of studies have demonstrated that the microbiota metabolites of dietary fiber are responsible for the benefits of dietary fiber, including improving fatty liver and other metabolic disorders 43,44. To test whether pectin alleviates fatty liver disease by the microbiota and its metabolites, FMT experiments were performed in the present study. The results demonstrated that heat-treated fecal slurry with 0.22 μm filtration had a more pronounced effect on alleviating MAFLD, indicating that microbial metabolites mediate pectin-induced health benefits. These results are consistent with emerging evidence that microbial metabolites and microbes synergistically regulate host metabolism.

A series of microbiota metabolites of dietary fiber in the colon, such as short-chain fatty acids, bile acids, and indole derivatives from tryptophan, have been reported to have beneficial metabolic functions. The findings of the present study revealed that pectin supplementation modulated intestinal microbiota-mediated tryptophan metabolism, specifically by promoting the degradation of tryptophan and the production of ILA. This pectin-induced metabolic shift resulted in elevated ILA levels in both the fecal samples and serum of the mice, which was also supported by a recent study utilizing an in vitro fermentation model cultured with human gut flora and several cohorts 13,21,45.

In recent years, a diverse array of indole derivatives has been demonstrated to possess a plethora of biological activities, including neuroprotective 46, anti-atherosclerotic 47,48, and antidiabetic nephropathy 49 activities. However, research regarding the protective effect of ILA on MAFLD is limited. In the present study, pectin intervention significantly increased hepatic bile acid synthesis from cholesterol and subsequent excretion to the gallbladder and feces, effectively reducing hepatic lipid accumulation. This effect was associated with increased ILA in the serum and hepatic CYP7A1 and FXR-BSEP activation. Using both in vivo and in vitro models, we demonstrated the role of ILA in alleviating fatty liver by activating hepatic CYP7A1 and FXR-BSEP expression. These results suggest a possible pathway in which pectin-mediated modulation of gut microbial communities enhances enterogenous ILA production from tryptophan. This, in turn, regulated bile acid metabolism through the upregulation of hepatic CYP7A1 and FXR-BSEP signaling.

FXR is expressed mainly in the liver and intestines 50. In the intestines, FXR activation reduces bile acid synthesis and intestinal reabsorption by regulating the expression of FGF19/21-CYP7A1, ASBT, IBABP, and OST α/β, promoting bile acid excretion into the venous system 51. In the liver, it serves as a critical nuclear receptor that modulates multiple metabolic pathways, including bile acid metabolism, lipid metabolism and glycol metabolism 52. However, clinical trials of related drugs have shown that extensive FXR activation might disrupt cholesterol homeostasis and prevent the metabolic conversion of cholesterol to BAs via downregulation of CYP7A1 expression 53.

In various animal studies, pectin interventions have been shown to either activate or inhibit hepatic FXR expression 54,55. Usually, hepatic FXR upregulation results in CYP7A1 inhibition 56. However, the present study revealed that pectin and ILA interventions activated hepatic FXR without inhibiting CYP7A1. Consistently, several recent studies revealed concurrent upregulation of hepatic FXR and CYP7A1 expression, including a study in which hyperoside was used to intervene in MAFLD mice 3, a study in which atorvastatin was used to intervene in HepB3 cells 57, and a study in which Peppermint oil was used to intervene in HepG2 cells 58. The expression of the CYP7A1 gene is subject to regulation by various factors, including gene polymorphisms, bile acid levels, dietary influences, hormonal factors, cytokines, pharmaceutical agents, and circadian rhythms. Furthermore, the regulation of CYP7A1 by FXR is indirect, and the molecules involved in this process can also be regulated by other factors. The observations in the present study suggest the possibility that alternative factors influence the upregulation of hepatic CYP7A1, which may counteract the inhibitory effect of FXR activation on CYP7A1 under multiple regulatory conditions. Alternatively, in a HepG2 high-fat cell model, we found that FXR suppression with siRNA could not entirely counteract the ILA upregulation of CYP7A1. These findings provide experimental evidence demonstrating an independent pathway by which ILA enhances CYP7A1 expression. However, further exploration is necessary to elucidate the specific mechanisms involved.

There are several limitations of this study that warrant consideration. First, although we used FMT, antibiotic-treated mice, and in vitro bacterial culture techniques to validate the important role of gut bacteria in this study, the use of gnotobiotic mice administered probiotics is needed to provide more scientific evidence of the direct link between specific bacterial strains and ILA. We will continue to conduct this research in the future. Second, the study focused on male mice to minimize hormonal variability, yet sex-specific differences in bile acid metabolism necessitate future investigations in female cohorts. Third, the translational relevance of murine and cell model findings to humans remains uncertain. The execution of clinical trials that assess the therapeutic potential of specific bacterial strains or ILA supplementation in patients with MAFLD is imperative in future work. In addition, the analysis of human MAFLD cohorts with public databases can also facilitate enhanced clinical translatability by investigating the correlation between *A. muciniphila* and *E. coli* and disease progression. This work will also be conducted in the future. Fourth, in this study, we did not conduct an in-depth investigation of the mechanism by which ILA regulates CYP7A1, which is independent of FXR. In the future, we plan to utilize liver and gut-specific FXR knockout mouse models to investigate this mechanism.

This study delineates a previously unrecognized axis linking citrus pectin, gut microbiota-derived ILA, and hepatic CYP7A1 and FXR-BSEP upregulation in mitigating MAFLD. By shifting tryptophan metabolism toward indole derivatives and enhancing bile acid synthesis and excretion, pectin addresses multiple facets of lipid dysregulation. These findings broaden the understanding of dietary fibers and position ILA as a potential therapeutic agent in the treatment of MAFLD.

## Supplementary Material

Supplementary figures and table.

## Abbreviations

ILA: indole-3-lactic acid
IPA: indole-3-propionic acid
MAFLD: metabolic-associated fatty liver disease
A. muciniphila: Akkermansia muciniphila
E. coli: Escherichia coli
CYP7A1: cholesterol 7 alpha hydroxylase
FXR: farnesoid X receptor
BSEP: bile salt export pump
ABCG5/8: ATP-binding cassette transporters G5/8
ARRIVE: animal research: reporting in vivo experiments
FMT: fecal microbiota transplantation
GAN: Gubra‒Amylin NASH
GPC: gel permeation chromatography
FTIR: Fourier transform infrared spectroscopy
OGTT: oral glucose tolerance test
MRI: magnetic resonance imaging
H&E: hematoxylin‒eosin
ORO: Oil Red O
OCT: optimal cutting temperature compound
NAS: NAFLD activity score
AASLD: American Association for the Study of Liver Diseases
TG: total triglycerides
TC: total cholesterol
HDL: high-density lipoprotein
LDL: low-density lipoprotein
ALT: alanine aminotransferase
AST: aspartate aminotransferase
TBA: total bile acid
RT-qRCR: real-time reverse transcription polymerase chain reaction
WB: western blot
SDS-PAGE: sodium dodecyl sulfate-polyacrylamide gel electrophoresis
PCA: principal component analysis
ASVs: amplicon sequence variants
FFA: free fatty acids
OA: oleic acid
PA: palmitic acid
BHI: brain heart infusion
PCA: principal component analysis
