# Supplementary material for: Citrus Pectin Supplementation Alleviated Hepatic Lipid Accumulation through Gut Microbiota Indole Lactic Acid Promoting Hepatic Bile Acid Synthesis and Excretion
Source: Int J Biol Sci. 2025 Jul 28;21(11):5015–33. doi: 10.7150/ijbs.116929 (PMC12374830; doi:10.7150/ijbs.116929)
Supplement: Supplementary file 1 — Supplementary figures and table. [file ijbsv21p5015s1.pdf]

Supplementary materials

(A)

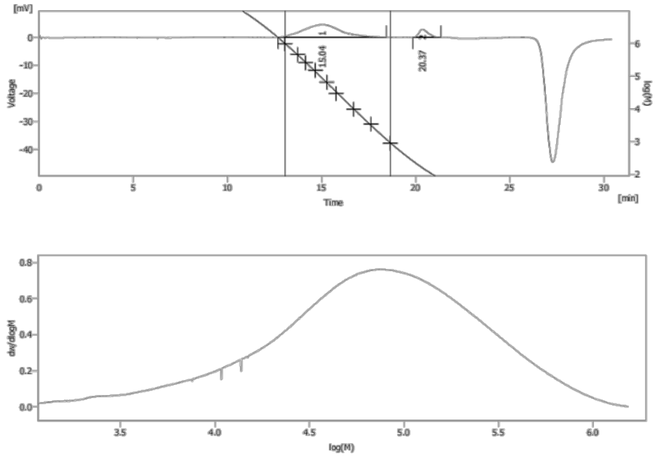

NO.	Max. RT	Initial RT	End RT	Mp	Mn	Mw	Peak height [mAU]	Peak area [mAU.s]	Peak area [%]
**1**	15.04	12.68	18.45	85572	32387	150392	4.42	611.68	86.71
**2**	20.37	19.85	21.34	155	139	146	2.79	93.79	13.29

(B)

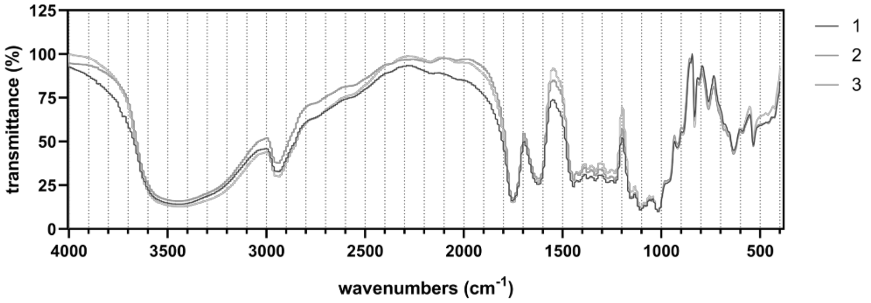

(C)

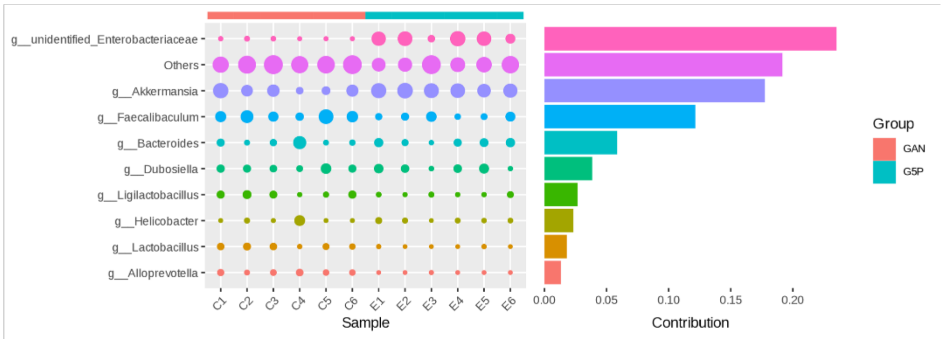

Figure S1. (A) pectin GPC chromatogram and main peak data; (B) pectin FTIR spectrogram; (C) the top ten contributing genera and their abundances to the divergence between the two groups

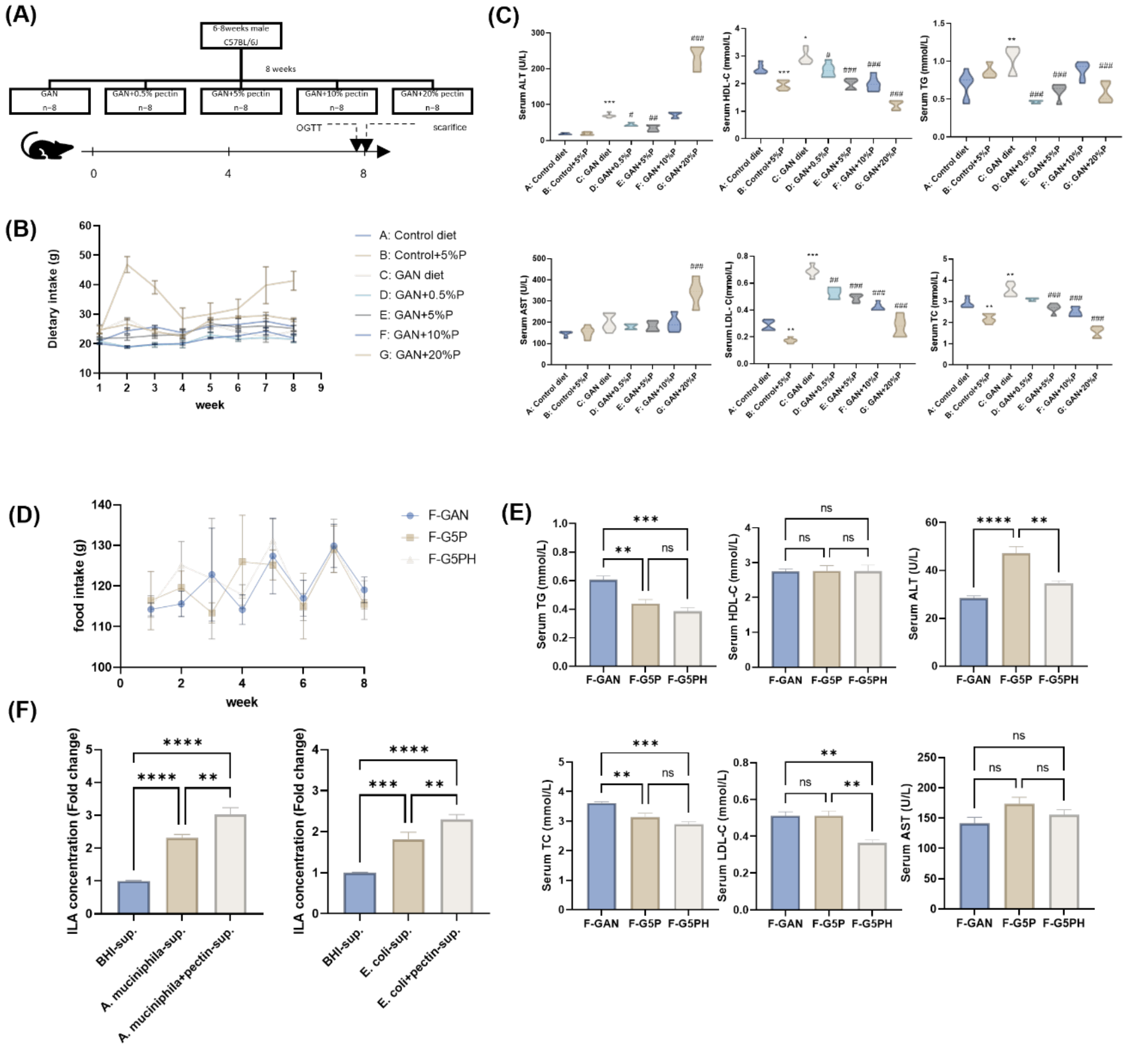

Figure S2. (A) schematic illustration of the design of animal experiment 1; (B) food intake of each group in animal experiment 1 during 8 weeks of treatment; (C) serum TG, TC, HDL-C, LDL-C, ALT, and AST levels of mice in animal experiment 1; (D) food intake of each group in animal experiment 2 during 8 weeks of treatment; (E) serum TG, TC, HDL-C, LDL-C, ALT, and AST levels of mice in animal experiment 2; (F) ILA levels of in vitro fermentation supernatants inoculated with *A. muciniphila* or *E. coli* with or without pectin supplement

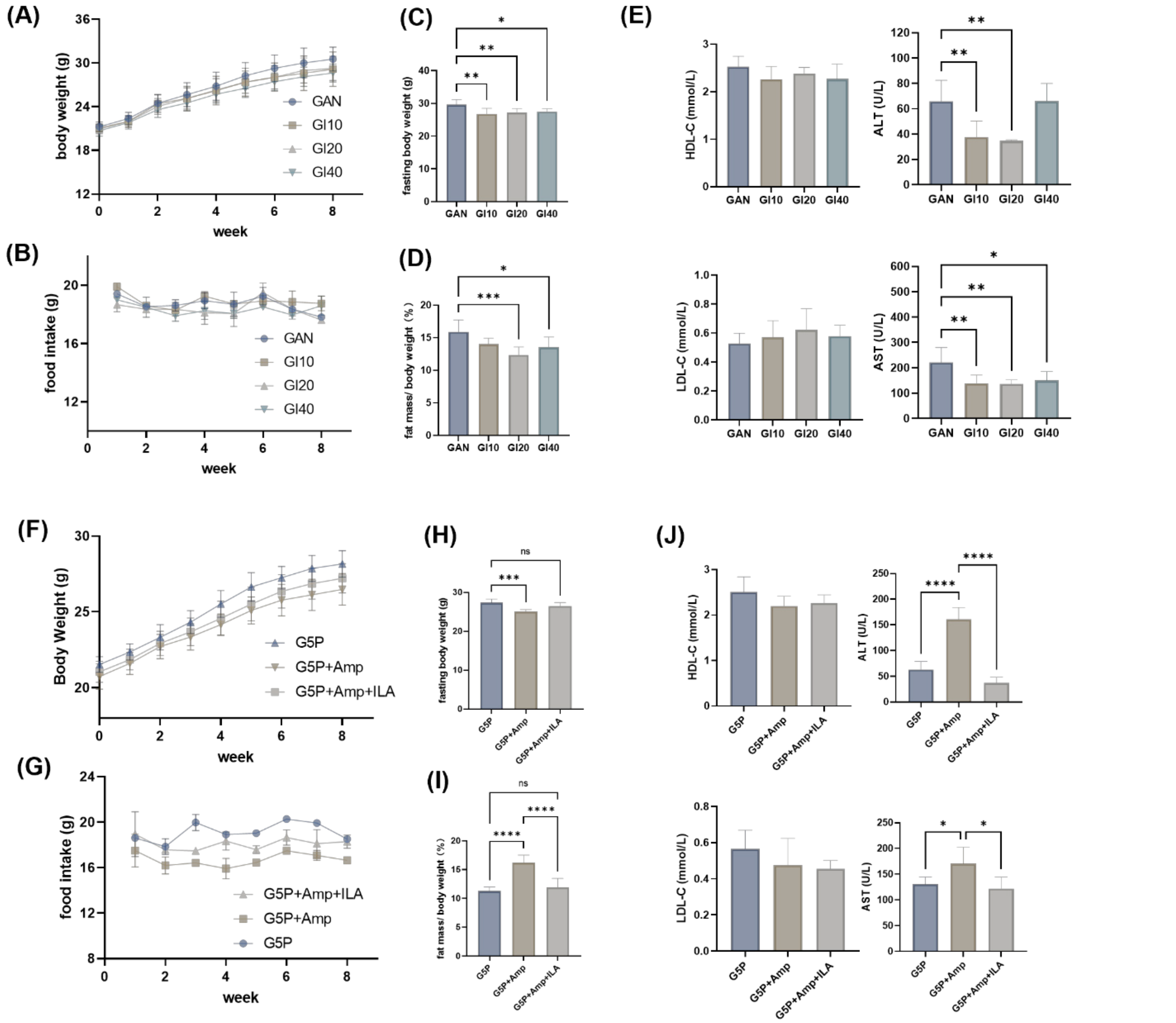

Figure S3. (A) body weight during 8 weeks of treatment; (B) food during 8 weeks of treatment; (C) fasting body weight at week 8; (D) fat mass level; (E) serum TG, TC, HDL-C, LDL-C, ALT, and AST levels; (F) body weight during 8 weeks of treatment; (G) food during 8 weeks of treatment; (H) fasting body weight at week 8; (I) fat mass level; (J) serum TG, TC, HDL-C, LDL-C, ALT, and AST levels

Table S1. Primer sequences used in this study

Gene	Forward primer sequence (5'-3')	Reverse primer sequence (5'-3')
SRB1	TGTACTGCCTAACATCTTGGTCC	ACTGTGCGGTTCATAAAAGCA
LDLR	TCAGACGAACAAGGCTGTCC	CCATCTAGGCAATCTCGGTCTC
FATP4	ACTGTTCTCCAAGCTAGTGCT	GATGAAGACCCGGATGAAACG
ABCA1	GCTTGTTGGCCTCAGTTAAGG	GTAGCTCAGGCGTACAGAGAT
ABCG5	AGGGCCTCACATCAACAGAG	GCTGACGCTGTAGGACACAT
ABCG8	CTGTGGAATGGGACTGTACTTC	GTTGGACTGACCACTGTAGGT
BSEP	TCTGACTCAGTGATTCTTCGCA	CCCATAAACATCAGCCAGTTGT
CYP27A1	CCAGGCACAGGAGAGTACG	GGGCAAGTGCAGCACATAG
CYP7A1	GGGATTGCTGTGGTAGTGAGC	GGTATGGAATCAACCCGTTGTC
ACAT1	CAGGAAGTAAGATGCCTGGAAC	TTCACCCCCTTGGATGACATT
ACAT2	CTATGAGGGCTATGCCTTGCC	GCTCAGCAGTAGTAACGAAGGA
HMGCR	AGCTTGCCCGAATTGTATGTG	TCTGTTGTGAACCATGTGACTTC
SREBP2	GCAGCAACGGGACCATTCT	CCCCATGACTAAGTCCTTCAACT
SREBP1	GCAGCCACCATCTAGCCTG	CAGCAGTGAGTCTGCCTTGAT
LXRα	CTCAATGCCTGATGTTTCTCCT	TCCAACCCTATCCCTAAAGCAA
FXR	GCTTGATGTGCTACAAAAGCTG	CGTGGTGATGGTTGAATGTCC
β-actin	GGCTGTATTCCCCTCCATCG;	CCAGTTGGTAACAATGCCATGT
